# Larval development in the Pacific oyster and the impacts of ocean acidification: Differential genetic effects in wild and domesticated stocks

**DOI:** 10.1111/eva.13289

**Published:** 2021-08-26

**Authors:** Evan Durland, Pierre De Wit, Eli Meyer, Chris Langdon

**Affiliations:** ^1^ Department of Fisheries and Wildlife and Coastal Oregon Marine Experiment Station Hatfield Marine Science Center Oregon State University Newport OR USA; ^2^ Department of Marine Sciences Tjärnö Marine Laboratory University of Gothenburg Strömstad Sweden; ^3^ Department of Integrative Biology Oregon State University Corvallis OR USA

**Keywords:** adaptation, domestication, genetics, ocean acidification, oysters

## Abstract

The adaptive capacity of marine calcifiers to ocean acidification (OA) is a topic of great interest to evolutionary biologists and ecologists. Previous studies have provided evidence to suggest that larval resilience to high *p*CO_2_ seawater for these species is a trait with a genetic basis and variability in natural populations. To date, however, it remains unclear how the selective effects of OA occur within the context of complex genetic interactions underpinning larval development in many of the most vulnerable taxa. Here we evaluated phenotypic and genetic changes during larval development of Pacific oysters (*Crassostrea gigas*) reared in ambient (~400 µatm) and high (~1600 µatm) *p*CO_2_ conditions, both in domesticated and naturalized “wild” oysters from the Pacific Northwest, USA. Using pooled DNA samples, we determined changes in allele frequencies across larval development, from early “D‐stage” larvae to metamorphosed juveniles (spat), in both groups and environments. Domesticated larvae had ~26% fewer loci with changing allele frequencies across developmental stages and <50% as many loci affected by acidified culture conditions, compared to larvae from wild broodstock. Functional enrichment analyses of genetic markers with significant changes in allele frequency revealed that the structure and function of cellular membranes were disproportionately affected by high *p*CO_2_ conditions in both groups. These results indicate the potential for a rapid adaptive response of oyster populations to OA conditions; however, underlying genetic changes associated with larval development differ between these wild and domesticated oyster stocks and influence their adaptive responses to OA conditions.

## 1 INTRODUCTION

As global oceans warm and become increasingly acidified, many marine species are threatened by environmental conditions that challenge or exceed their physiological limits (Doney et al., 2012). For sessile species that cannot migrate to favorable habitats, physiological plasticity may provide short‐term acclimation to environmental stressors but long‐term survival of vulnerable species will likely have to rely on genetic adaptation (Hoffmann & Sgrò, 2011). The adaptive capacity of a population depends on many intrinsic and external factors but is fundamentally based on its standing genetic diversity from which new combinations of traits can arise (Munday et al., 2013). Understanding how marine species will adapt to ocean acidification (OA) is of critical importance in order to better understand potential impacts of future ocean conditions on marine ecosystems.

Marine invertebrate species are well suited to studies on how changes in ocean conditions may drive adaptive responses: They are typically genetically diverse (Bazin et al., 2006), highly fecund (Llorda, 2002), and sensitive to environmental stressors, especially during larval development (His et al., 1999). Marine calcifiers, such as bivalves and echinoderms, are also highly vulnerable to OA, which is one of the primary ways that climate change is affecting marine ecosystems (Doney et al., 2009). These attributes of marine invertebrates allow for rapid adaptive responses to selection, with significant genetic changes in larval populations detected within a single generation (Bitter et al., 2019; Brennan et al., 2019; Pespeni et al., 2013).

One of the challenges for identifying genetic components of fitness for a specific environmental stressor like OA in many of the most sensitive marine species is that larval development is often a protracted and complex physiological process. Consequently, larval sensitivity to acidification stress may vary across developmental stages and involve a range of different physiological responses (Kapsenberg et al., 2018). For instance, shell formation in early developing bivalve larvae is acutely hindered due to the reduced saturation state of aragonite (Ωarag; Waldbusser et al., 2015a) which frequently accompanies OA conditions. Later in larval development, however, additional physiological processes, such as respiration, feeding, and metabolism, can be negatively impacted by other properties of acidified seawater, such as elevated *p*CO_2_ and reduced pH (Pan et al., 2015; Waldbusser et al., 2015b). Developmentally targeted gene expression studies are capable of identifying discrete physiological responses to stress (e.g., De Wit et al., 2018; O’Donnell et al., 2010; Wang et al., 2017), but genetic changes in the larval population, that may or may not occur in response to these stresses, accumulate throughout development and may be difficult to differentiate in surviving individuals that are sampled only once at the end of the developmental period.

Another aspect that complicates detection and interpretation of genetic “outliers” in many bivalve species is that larval survival is characteristically very low, with only a small proportion of individuals surviving from fertilization to the postmetamorphic juvenile (spat) stage (type III survivorship). This high larval mortality is reported to be due to high numbers of negative or deleterious alleles (genetic load) present in both selectively bred and wild populations of many marine invertebrates (Plough, 2016); for example, Plough et al. (2016) estimated that 11–19 deleterious mutations in wild stocks of Pacific oysters (*Crassostrea gigas*, Thunberg 1793) rendered >95% of all larval progeny genetically inviable. In this context, it is apparent that estimates of the adaptive capacity of marine invertebrates to environmental stressors, such as OA, require consideration of both the genetic effects of larval development inherent to that species and population, as well as those attributable to external environmental conditions.

A previous set of experiments compared the relative performance of larval Pacific oysters spawned from domesticated stocks to those of their ancestral naturalized “wild” population in the Pacific Northwest USA (Durland et al., 2019). In that study, across two spawns and years, domesticated oysters produced spat with significantly greater growth and survival in both ambient and high *p*CO_2_ conditions. This level of consistent difference across both environments was surprising, given that only ~5 generations of controlled breeding separated these two populations and larval traits were not intentional targets of selection in the domesticated oyster stock.

In this study, we evaluated the extent to which the genetic composition of larval populations, derived from domesticated and wild broodstocks, changed when reared under ambient (~400 *p*CO_2_) and acidified (~1600 *p*CO_2_) seawater conditions. We used pooled sequencing of genomic DNA from larvae and spat sampled at 2 and 22 days postfertilization to test three hypotheses comparing these two populations: (1) Relative differences in overall survival through metamorphosis correspond with different amounts of genetic change; (2) larval development in high *p*CO_2_ conditions have a greater genetic effect on wild than domesticated larval populations; and (3) genetic changes in both larval populations in each environment are similar in identity (SNPs in common) and function (genes or Gene Ontology). Collectively, these hypotheses were formulated to investigate the adaptive capacity of *C. gigas* to OA conditions, against a background of genotype‐dependent mortality in these two populations.

## 2 METHODS

### 2.1 Broodstock and conditioning

The experiment was conducted in 2015 as part of a broader comparison of the phenotypic effects of high *p*CO_2_ seawater on larvae created from selectively bred oysters from the Molluscan Broodstock Program (MBP; de Melo et al., 2016) and wild oyster stocks, as described by (Durland et al., 2019). Briefly, in the spring of 2015, *n* = 400 oysters from the 20 top‐performing families from MBP (ranked based on farm yields; de Melo et al. (2016)) and *n* = 60 wild oysters from Willapa Bay, WA, were transferred from the broodstock repository at the Hatfield Marine Science Center (HMSC), Yaquina Bay, Oregon, to conditioning tanks. Broodstock was provided with flow‐through seawater (~2 L min^−1^) and a mixed algal diet of 50/50 (by cell concentration) *Isochrysis galbana* (C‐iso) and *Chaetoceros gracilis* at approximately 20–30,000 cells ml^−1^. Water temperature was increased from ambient (11°C) to 20°C over the course of two weeks and maintained at this temperature for 25 additional days to facilitate gonad development prior to spawning.

### 2.2 Cross design and spawning

For both MBP and wild broodstock groups, 95 single‐pair matings (1♀ × 1♂) were created from available parents. A total of 19 male and 19 female oysters from MBP families were crossed in a semi‐factorial fashion: One male and one female oyster from each family were individually paired with 4–6 individuals (of opposite sex) from other MBP families with a low coefficient of co‐ancestry (<10%; de Melo et al., 2016) with no reciprocation. Wild broodstock had a heavily skewed sex ratio (~ 10:1 female to male), and thus, 95 crosses were made from 19 females and 5 males in a fully factorial mating design (every male paired with every female). For each cross, eggs and sperm were manually removed from ripe parents (strip spawned) and suspended in seawater. The total number of eggs from each female was counted and divided equally into 4–6 replicate beakers and fertilized with sperm suspensions from different males. After 1 h, fertilization was confirmed visually and eggs were washed of excess sperm on a 25 µm screen. Fertilized eggs were proportionally combined to form two composite embryo pools (one each for MBP and wild) that contained approximately equal proportions of each of the 95 crosses.

### 2.3 Seawater manipulation and sampling

Ambient (~400 μatm CO_2_, pH = 7.9–8.1, Ω_arag_ = 2.3–2.7) and high *p*CO_2_ (~1600 μatm CO_2_, pH = 7.5–7.6, Ω_arag_ = 0.9–1.2) seawater treatment conditions were created by filling two identical 200‐L tanks with seawater (25°C, salinity 32, 10 μm‐filtered) and equilibrating them overnight via vigorous aeration with outside air. High *p*CO_2_ treatment water was then aerated for several hours with a gas mixture of CO_2_‐stripped air and pure CO_2_ to result in a final *p*CO_2_ concentration of ~1600 μatm. This *p*CO_2_ level was selected to approximate naturally occurring concentrations of CO_2_ experienced at hatcheries on the West coast USA during upwelling events (Barton et al., 2012). Gas mixing was controlled by paired mass flow controllers (Alicat, Tucson, AZ): one each for air and CO_2_. Culture units consisted of 10‐liter polycarbonate chambers (BearVault, San Diego, CA) fitted with a sealing lid, and silicone ring seal (McMaster‐Carr, Santa Fe Springs, CA). No supplemental aeration was supplied to the larval rearing units throughout the experimental period.

The pH, temperature, and dissolved oxygen of individual culture chambers were monitored daily: Nominal pH and temperature values were measured with an Orion Star A11 pH meter (Thermo‐Fisher USA) with a Ross Ultra pH/ATC triode, calibrated with NIST buffers, and adjusted with a seawater standard (Batch 22, A.G. Dickson, Scripps Institution of Oceanography, USA). Dissolved oxygen was measured with a YSI 85 meter (YSI, Yellow Springs, OH, USA). The temperature and dissolved oxygen content of culture units was stable throughout larval rearing, with averages (± standard error) of 24.5°C (±0.15) and 8.15 mg L^−1^ (±0.03), respectively.

Seawater samples for carbonate analysis were also obtained from seawater used to fill the chambers and after 48 h of culture (before each water change) to account for changes in chemistry arising from off‐gassing or respiration. These samples were stored in gas‐tight 350‐ml amber glass bottles, poisoned with 30 µl of saturated mercuric chloride (HgCl_2_) solution, and sealed with a metal crimp cap for later analysis. Samples were analyzed at the laboratory of Dr. Burke Hales at Oregon State University, following the procedure outlined by Hales et al. (2005) and Bandstra et al. (2006) to obtain values for sample total dissolved carbon dioxide (TCO_2_), *p*CO_2_, and seawater pH, from which Ω_arag_ and Ω_calc_ values were calculated. These parameters varied somewhat throughout the larval culture period, but remained reasonably within an acceptable range for each treatment. A summary of seawater carbonate chemistry of the experimental treatments is found in Figure S1 and detailed data are in File S1.

### 2.4 Larval culture

Approximately 5 h after fertilization ~200,000 embryos from MBP or wild pools were distributed to each culture replicate, resulting in an effective stocking density of 20 embryos ml^−1^. Culture units were filled with either ambient (~400 μatm) or high (~1600 μatm) *p*CO_2_ seawater. Each treatment level (broodstock × water type) was replicated five times, for a total of 20 culture units. Water was changed every 48 h by sieving larvae on a mesh screen, filling the culture units with fresh seawater equilibrated to treatment *p*CO_2_ levels, and re‐stocking the larvae. Antibiotics were added prophylactically in order to reduce bacterial respiration in culture units that would unduly affect seawater carbonate chemistry (Waldbusser et al., 2015a). Antibiotics were alternated at each water change between a mixture of chloramphenicol/ampicillin (2 ppm and 10 ppm, respectively) and 20 ppm streptomycin to reduce the risks of development of resistant bacterial strains.

Microalgal diets were supplied once daily, starting 2 days postfertilization (dpf), beginning with C‐iso (*Isochrysis galbana*) at 20,000 cells ml^−1^ and increasing the ration by 5000 cells ml^−1^ day^−1^. The diatom species *Chaetoceros gracilis* was gradually incorporated into the diet on day 4, starting at 5% (based on cell concentrations) until it accounted for 50% of the algal diet by day 11, where it was maintained for the duration of the experiment. Larval densities were reduced to 10 ml^−1^ on day 2, 5 ml^−1^ on day 6, and 1 ml^−1^ at the pediveliger stage at day 14. Larval density was reduced randomly, with no selection for larval size, and maintained in this fashion to provide equal and optimal environments for growth and survival and to limit respiratory contributions to seawater *p*CO_2_ levels. One ambient *p*CO_2_ culture unit suffered complete larval mortality on day 10 but all prior data points and samples were retained for analysis. After the appearance of eyed larvae (indicating a readiness to metamorphose), larvae were screened on a 240 μm sieve to retain pediveliger larvae which were subsequently induced to metamorphose by exposure to 1.8E10^−4^ M epinephrine for 2 h (Coon et al., 1986). The larvae that passed through this screen were returned to the appropriate culture unit. After epinephrine exposure, the pediveliger larvae and newly metamorphosed “spat” were rinsed in seawater and returned to the culture vessel with the remainder of the larval group. Metamorphosis was induced in this fashion on days 16, 18, and 20. The experiment was terminated on day 22 after the majority of potentially competent “eyed” larvae had metamorphosed.

### 2.5 Larval sampling

**FIGURE 1:**
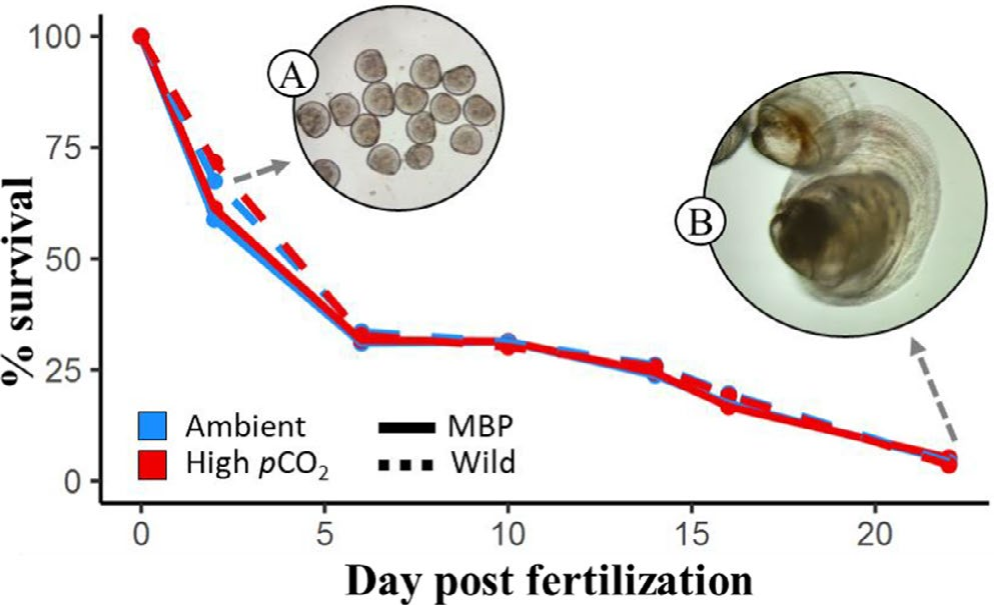
Survival of larvae from MBP (solid lines) and wild (dashed lines) groups reared in ambient (blue) and high (red) *p*CO_2_ seawater. Samples of D‐larvae at 2 dpf (a) and spat and eyed pediveliger larvae at 22 dpf (b) were obtained for DNA extraction and sequencing

Larval survival was estimated from counts of live larvae in each culture unit on days 2, 6, 10, 14, 16, and 22. Sampling and counting methods are described in detail by Durland et al. (2019). Briefly, at each time point larvae were concentrated in 250‐ml beakers, subsampled, and enumerated, and survival was calculated as the cumulative percent of live larvae remaining from the embryos initially stocked in the containers, after correction for density thinning and sampling. Morphometric samples were preserved in vials filled with 10 ml seawater and 200 μl of 10% buffered formalin (pH = 8.1–8.3). Samples for DNA extraction were obtained at days 2 and 22 with ~3000 and ~200 individual oysters per culture unit for 2 and 22 dpf, respectively (Figure 1), and preserved in 95% ethanol. Samples from day 22 were a composite of metamorphosed spat as well as eyed larvae that were retained on a 240 μm screen.

### 2.6 DNA extraction and library preparation

From each sample, genomic DNA was extracted using a CTAB extraction method with RNAse treatment detailed by (Panova et al., 2016). DNA concentration and purity were assessed using a Qubit fluorometer (Thermo Scientific). 2b‐RAD libraries were prepared following a modified protocol based on Wang et al. (2012), using the BcgI restriction enzyme. This protocol can be found at https://github.com/E‐Durland/oyster‐poolseq. All individual samples (*n* = 39) were given unique barcodes and pooled in sets of ~32 samples per sequencing lane. Single‐read, 50 base pair (bp), target‐length sequencing was conducted on an Illumina HiSeq2500 platform in the SNP&SEQ Technology Platform at the National Genomics Infrastructure at Uppsala University, Sweden.

### 2.7 Bioinformatics

The bioinformatic analysis of DNA sequences followed a 2b‐RAD‐specific pipeline constructed by Dr. Eli Meyer (v3.0) and has been uploaded, along with custom scripts, at the github page referenced previously. Briefly, de‐duplexed raw reads were truncated to 36 bp in length and quality filtered to include only reads that had <10 bp with phred quality scores <20. The remaining reads were further filtered for adaptor sequence contaminants using BBDuk (part of the BBtools package; Bushnell (2014)) and a kmer size of 12. Cleaned, high‐quality reads were mapped to the reference genome (Zhang et al., 2012; the only available reference genome at time of analysis) using the SHRiMP software package (Rumble et al., 2009) with default mapping parameters but allowing for a maximum of three genomic alignments per read and retaining the single best alignment. Alignments were then filtered to retain only those with ≥30 bp matching the reference sequence. At the end of these steps, the files contained an average of 1.02 × 10^6^ reads across ~2.6 × 10^5^ loci for an average coverage depth of ~4× across all loci. Sequence alignment map (SAM) files for each sample were then converted to tab‐delimited files with read counts of each nucleotide at each locus, and the entire dataset was merged. A custom Python script was used to then remove reads with a sequencing depth of <50 or >1000 (Schlötterer et al., 2014) and loci with >2 alleles observed. Polymorphic loci were filtered to one SNP per 36 bp tag to limit complications arising from potential ambiguities in mapping of the reads which may erroneously result in the detection of multiple polymorphisms in close proximity to one another. These filtering steps left 8514 SNPs for downstream analyses.

To compare changes in allele frequencies, we interpreted read counts directly from nucleotide frequencies in each pool (replicate) in the output file of SAMBasecaller.pl. All quality filtering and mapping statistics can be found in Supplementary Information (Table S3). All bioinformatic analyses were conducted on computing infrastructure at the Center for Genomic Research and Biocomputing (CGRB) Core Laboratories at Oregon State University.

### 2.8 Data analysis

#### 2.8.1 Measuring genetic diversity and differentiation

Group‐level differences in genetic diversity and overall differentiation were analyzed by evaluating allele frequency spectra, nucleotide diversity (π), and pairwise *F*_ST_ values on a SNP‐by‐SNP basis for the 1288 polymorphisms that were retained from quality filtering (see below). Nucleotide diversity was calculated manually with methods adapted from Begun et al. (2007). Pairwise *F*_ST_ was also calculated manually, and bootstrap confidence intervals were estimated with 1000 re‐samplings of the full dataset. Allele frequency spectra of the two populations were compared using a Kolmogorov‐Smirnov (K‐S) test. Allele frequencies of each of the five replicates from MBP and wild larval pools were also analyzed with principal components analysis (PCA) using the “prcomp” function in R (R Core Team, 2015).

#### 2.8.2 Detecting changes in allele frequencies

Loci in this dataset were filtered for missing data and rare polymorphisms, keeping only loci for which three or more replicates per treatment level (broodstock x treatment x time) had reads present and with a starting minor allele frequency (MAF) in at least one broodstock group of >1%. This left 1288 SNPs for statistical modeling. The read count data for each allele at each remaining locus were analyzed with a binomial generalized linear model (GLM) with the formula: $\left(A_{1}:A_{2}\right)∼\;\beta_{\mathrm{Stage}}+\beta_{\mathrm{Trt}}+\left(\beta_{\mathrm{Stage}}\;\times \;\beta_{\mathrm{Trt}}\right)$ where A_1_ and A_2_ refer to the read counts of alternative alleles at each locus and $\beta_{\mathrm{Stage}}$ and $\beta_{\mathrm{Trt}}$ represent estimates for developmental stage (2 or 22 dpf) and treatment (seawater *p*CO_2_), respectively. Initially, these data were modeled with broodstock type (MBP vs wild) as a third fixed effect (and corresponding interactions) but this resulted in an overabundance of two and three‐way interactions that (1) complicated the interpretation of results and (2) effectively over‐fit variation on many loci that were data poor. Analyzing the effects of development and seawater treatment on genetic changes in larval pools of each broodstock group separately also allowed for a more robust detection of significant change arising from the standing genetic variation of each pool. The model for each locus compared read counts of both alleles (*A*_1_/*A*_2_) in each of five replicates at each of the four levels of the model (stage (2) × treatment (2)). A visualization of the variance among replicates and populations can be found in Figure S2. For each locus, in both the MBP and wild populations, *p*‐values were obtained from the type III sum of squares of the GLM and mean allele frequencies of each treatment level were calculated. *p*‐values were adjusted with a Benjamini‐Hochberg false discovery rate correction (*p* < 0.05). All data analyses were conducted in R (R Core Team, 2015), and figures were plotted with the “ggplot2” package (Wickham, 2016).

#### 2.8.3 Mapping markers to linkage groups

For analyses of genetic change relative to the architecture of the genome, loci were assigned to linkage groups (LGs) by comparing genomic mapping locations to markers on a previously published linkage map (Hedgecock et al., 2015). To accomplish this, we used the reference genome (Zhang et al., 2012) as a “bridge” to the linkage map in a method conceptually similar to that used by Sutherland et al. (2016). For each SNP in our dataset that shared a genomic scaffold with a “mapped marker” from the linkage map, (1 SNP: 1 mapped) was assigned to the same position as the reported mapped marker. If multiple SNPs from our dataset were found on a scaffold that was represented by single a mapped marker (≥2 SNPs: 1 mapped), all the corresponding SNPs from our dataset were assigned to the (same) genomic position of the mapped marker. When multiple mapped markers were found on the same scaffold, SNPs in our dataset were assigned the linkage position of the marker that was nearest in the scaffold (by base pair location). Loci existing on genomic scaffolds which were not found on the linkage map or scaffolds which appeared on multiple linkage groups were omitted from this step of the analysis. In total, *n* = 334 markers (~25%) were mapped to linkage groups in this way (see Files S2–S3). For the remainder of this paper, we will refer to these as “mapped loci.”

#### 2.8.4 Functional analyses

Lastly, for functional analyses, genomic regions within 5kb up and downstream of each mapped locus were searched for gene annotations in the reference genome (Zhang et al., 2012), yielding 331 genes in total. This list of genes was then entered into the UniProtKB database (www.uniprot.org) which provided putative functions and gene ontology (GO) terms for 204 of the genes predicted in the annotation. Many genes are assigned with multiple, partially overlapping, GO terms resulting in 483 unique GO IDs for this gene set. The entire gene list, and their associated GO terms, was then analyzed with a gene score resampling procedure (GSR) in ErmineJ (Gillis et al., 2010), which compared GO terms associated with genes near markers that were significantly changed by developmental stage or seawater treatment in each broodstock group to GO terms stemming from genes in proximity to markers that were unchanged. This technique does not strictly discriminate on *p*‐value thresholds (e.g., *p* < 0.05) but, instead, incorporates *p*‐values as a continuous measure of significance and evaluates functional over‐representation of specific GO terms from the entire dataset. We conducted GSR analyses for *p*‐values from each of the main effects (“Stage” and “Trt”) and those in the overlapping category (“Stage + Trt”; Table 1) were used in both tests. We also performed the test on the *p*‐values for interaction effects (“Stage * Trt”) but, in this case, no functional categories were over‐represented and so the results were not included.

**TABLE 1:** Cross‐tabulation of the number of SNPs with significantly different minor allele frequencies (*p* < 0.05) by effect type in MBP and wild larval oyster groups

		Wild					
		None	Stage	Trt	Stage + Trt	Stage * Trt	Total
**MBP**	**None**	855	131	46	40	26	1098
	**Stage**	60	4	9	15	19	107
	**Trt**	13	4	3	3	0	23
	**Stage + Trt**	4	0	0	1	2	7
	Stage * Trt	26	6	6	6	9	53
	**Total**	958	145	64	65	56	1288

Note

“Stage” refers to changes detected between developmental states of “D‐larvae” at day 2 and eyed larvae and spat at day 22. Treatment (Trt) refers to changes detected between ambient and high *p*CO_2_ seawater culture environments. “Stage + Trt” and “Stage * Trt” refer to additive and interactive effects, respectively, of both variables.

## 3 RESULTS

### 3.1 Genetic diversity comparisons

By comparing allele frequencies from day 2 samples from ambient conditions (using quality filtered SNPs but including rare alleles <1% MAF), we found that MBP and wild larval groups were moderately differentiated (mean pairwise *F*_ST_ = 0.0297) which is consistent with recent estimates from wild and farmed stocks of *C. gigas* (Sutherland et al., 2020). Wild larvae had slightly higher mean allele frequency (0.074) compared to MBP (0.072) as well as nucleotide diversity (mean π = 0.108 and 0.106, respectively). While the overall differences between the stocks were small, wild larvae did have a relatively greater abundance of rare alleles (MAF < 10%), which significantly skewed the composite density function of this distribution (K‐S test *p*‐value = 0.004). Comparisons of genetic diversity and differentiation can be found in Figure S3.

### 3.2 Survival and development

All phenotypic measurements and analyses (survival and growth development) for this study were previously reported by Durland et al. (2019). A detailed description and analysis of the relative performance of MBP and wild larvae in these treatment conditions for the cohort used in the current study (year 2015) can be found therein. Several key metrics pertinent to the genetic analysis of this study and are reported here. At 48 h postfertilization, larval survival was not significantly different between MBP or wild groups or between ambient and high *p*CO_2_ seawater (*p* > 0.05). The proportion of fully formed “normal” D‐hinge larvae at day 2, however, was ~13% lower in both groups cultured in high *p*CO_2_ seawater (*p* < 0.05; Figure S4). Larval growth and survival through the remainder of veliger stages (to day 16) were statistically uniform among all cultures. By day 22, pediveliger larvae (>240 µm) and spat were more abundant in MBP $\left(\bar{x}=3482\right)$ than wild $\left(\bar{x}=1914\right)$ groups, representing an ~82% increase (Figure S4; statistical analysis in Durland et al. (2019); Table S15). Metamorphosed spat from MBP larval groups $\left(\bar{x}=582\;\mu m\right)$ were also ~16% larger than wild counterparts $\left(\bar{x}=501\;\mu m\right)$; Durland et al. (2019); Table S17A. The total survival values of all larvae (all size/developmental stages) were not significantly different between MBP and wild populations (Figure 1; Figure S4), owing to a greater abundance of small, underdeveloped veliger larvae (<240 µm) in the latter group. Larvae that had not reached pediveliger stage after 22 days of development in hatchery conditions were unlikely to do so (Coon et al., 1990) and, thus, not included in performance metrics or genetic samples. In this experiment, high *p*CO_2_ seawater conditions did not have any significant effect on overall growth, survival, or metamorphic success in either group (*p* > 0.05; Figure S4; see Durland et al. (2019) for more details).

### 3.3 Genetic effects of larval development and seawater treatment

**FIGURE 2:**
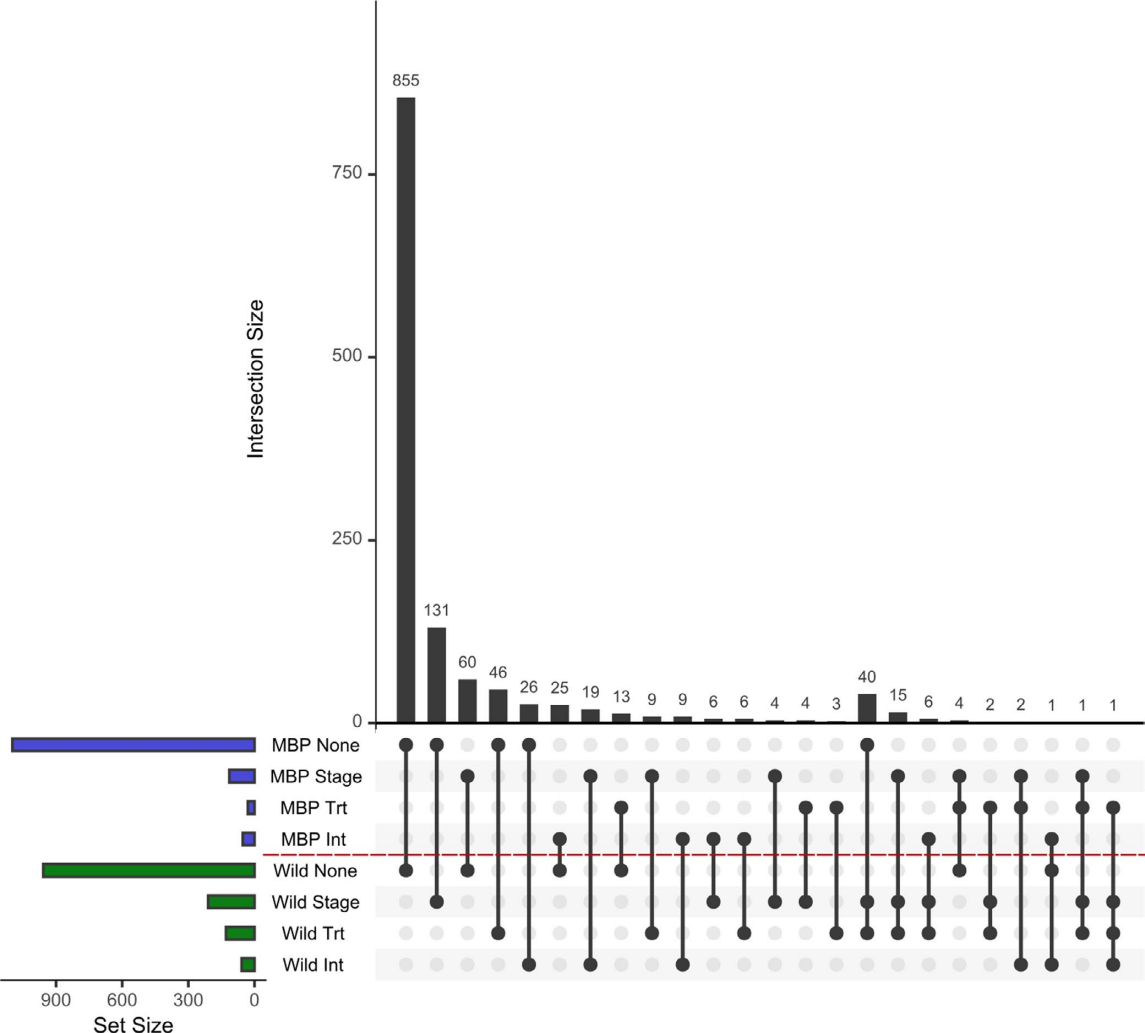
Upset plot of cross‐classification of SNPs changing by category in Table 1. Vertical barplot represents total number of SNPs in each shared category. Points and lines in the matrix visualize these connections (e.g., MBP none and Wild none), and the colored horizontal bars are the total number of SNPs in each category (none, stage, treatment, or interaction), by group (MBP or wild)

Among the 1288 SNPs available for analysis, 66% (*n* = 855) exhibited no significant change in allele frequency owing to larval development or seawater treatment in either MBP or wild larval groups. Using type III sums of squares from GLMs on each locus, we classified the remaining ~34% of significantly changed SNPs (*n* = 433) into the exclusive categories: Stage (developmental period), Treatment (Trt; seawater *p*CO_2_), Stage and Trt (Stage + Trt), or an interaction between these factors (Stage * Trt). The source of genetic changes in each group (e.g., “none,” “Stage,” “Trt”) was statistically different between groups *X* ^2^ (*df* = 1, *n* = 1288) = 9.53, *p* < 2.2 × 10^−16^) indicating that genetic changes were not similarly attributable to each category in MBP and wild larval populations (Figure 2). From a quantitative perspective, wild larval pools had ~74% more SNPs with significantly altered allele frequencies overall (*n* = 330 or ~25.6% of the total) than MBP larvae (*n* = 190 or ~14.8% of the total; Table 1; Figure 2).

**FIGURE 3:**
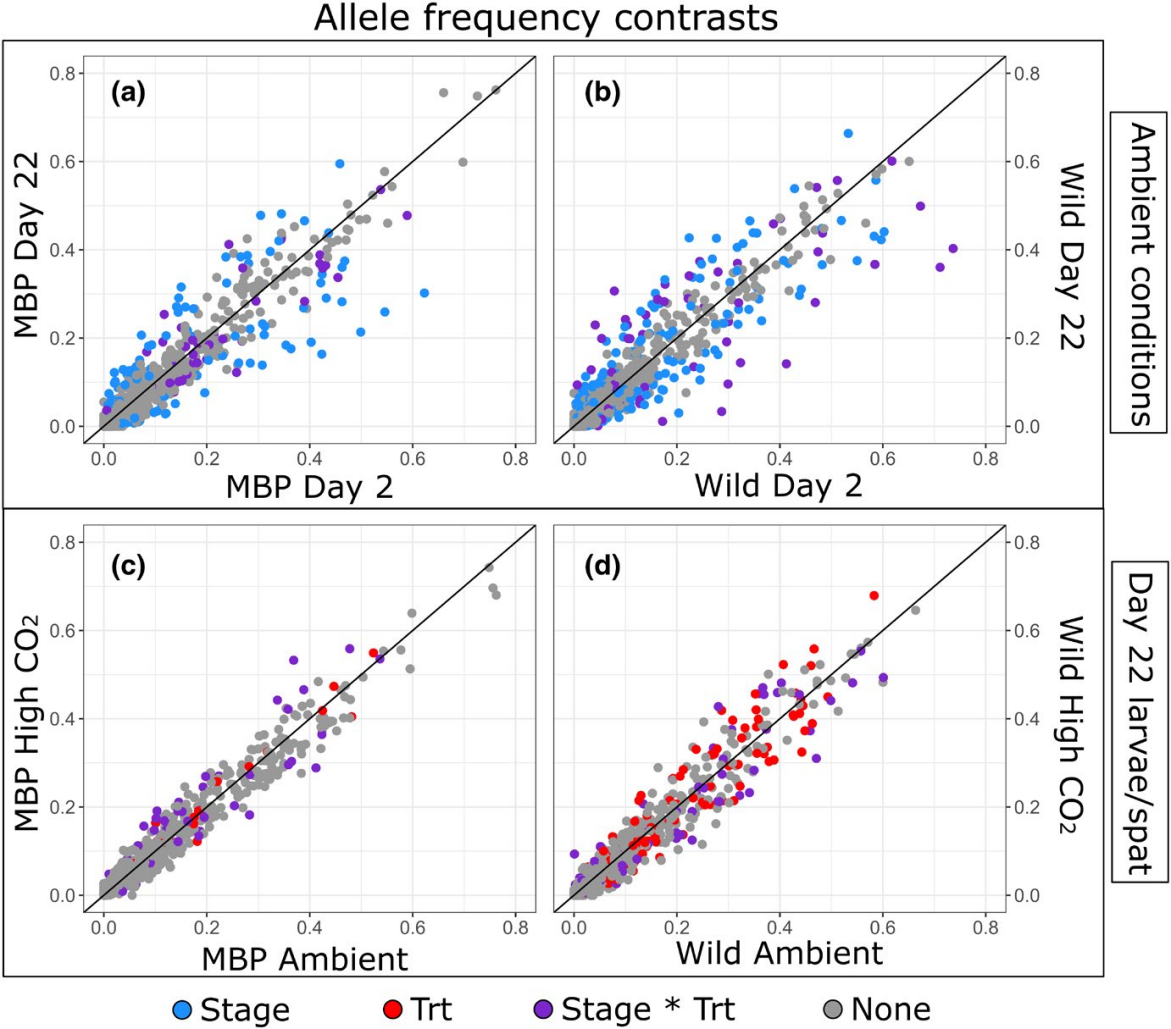
Changes in allele frequencies owing to development and seawater *p*CO_2_. A comparison of minor allele frequencies in MBP (a) and wild (b) larval groups between day 2 and 22 in ambient conditions and between spat/larval populations (at day 22) for MBP (c) and wild (d) larvae reared in ambient and high *p*CO_2_ treatments. Point color corresponds to significant changes (*p* < 0.05) based on effect: blue for “Stage,” red for seawater treatment (“Trt”), and purple for an interaction of the two (“Stage * Trt”). Gray points represent SNPs for which no significant change in MAF was detected

For both broodstock types, larval development had the greatest influence on allele frequency changes, affecting MAFs for 107 and 145 SNPs in MBP and wild groups, respectively, resulting in a ~26% greater number of loci with altered allele frequencies between day 2 and day 22 in the wild group, independent of treatment effects. The effects of high *p*CO_2_ seawater treatment on genetic changes in larval groups were even more different between the two larval populations. Wild larval pools had *n* = 185 SNPs with altered allele frequencies owing to high *p*CO_2_ seawater compared to *n* = 83 found in MBP groups, representing a ~223% difference. Seawater treatment effects also additively overlapped with “Stage” effects (Stage + Trt) more so in wild (*n* = 65) than MBP (*n* = 7) groups but interactive effects between development and treatment (Stage * Trt) were approximately equally abundant in MBP (*n* = 53) and wild larvae (*n* = 56). Among all the loci with significantly affected allele frequencies, there were very few that were shared between the groups (Table 1). Allele frequency changes are visualized in Figure 3, comparing the average allele frequency of each SNP (among replicates) at 2 and 22 dpf in ambient conditions (Figure 3a,b), as well as genetic differences between day 22 populations in each broodstock group reared in ambient and high *p*CO_2_ conditions (Figure 3c,d).

**FIGURE 4:**
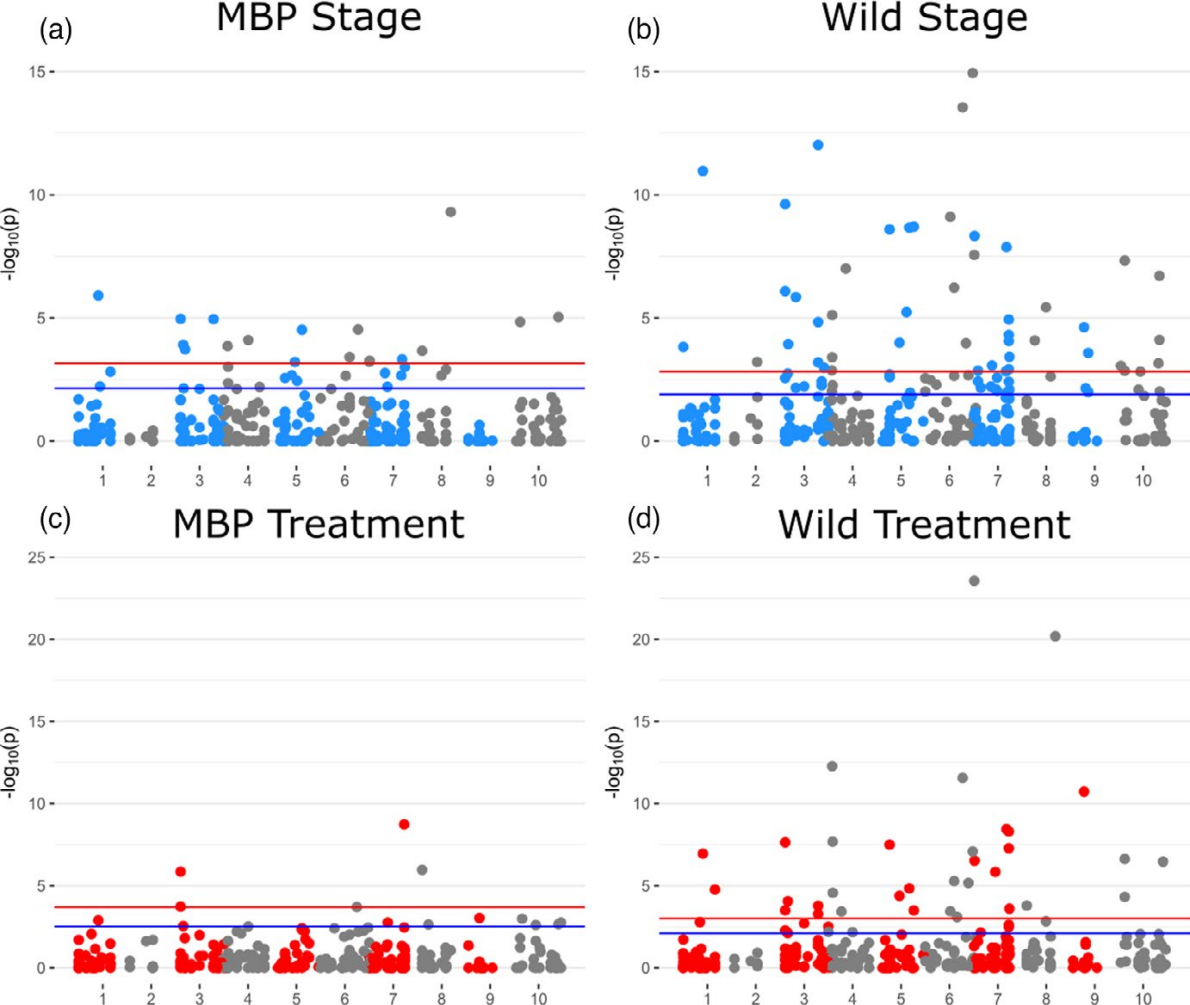
Manhattan plots of *n* = 334 mapped SNPs and the −log10 (*p*‐value) of significance for change in allele frequencies owing to developmental “Stage” (blue; a&b) and seawater treatment (red; c&d) effects. Red and blue horizontal lines on each plot reflect genome‐wide significance thresholds of *p* = 0.05 and *p* = 0.01, respectively, after a Benjamini‐Hochberg correction (FDR = 0.05)

The assignment of loci to linkage groups via scaffold association resulted in a significant reduction of the size of the dataset but the remaining 334 SNPs, for which genomic positions could be approximated, provide moderate coverage on each of the 10 linkage groups. Manhattan plots were used to assess the scale of genetic change of each of the primary factors (Stage and Trt) across the genome in both MBP and wild larval groups (Figure 4). These plots highlight the increased abundance and significance of genetic changes in wild groups, relative to MBP, over larval development in general (Figure 4a,b) and when cultured in high *p*CO_2_ seawater (Figure 4c,d). Loci with significant MAF changes occur across the genome in a relatively homogenous fashion, without any clear clustering in specific regions. It is worth noting that the way in which these markers were assigned positions on linkage groups resulted in several cases where numerous markers occupied the same approximate genomic position, based on scaffold associations on the reference genome (see Methods). In these instances, such as at the tail‐end of LG 7, a cluster of outlier loci may seem to represent a genomic region of interest, but this finding should be interpreted with caution (see Discussion).

### 3.4 Functional enrichment

Functional enrichment analyses, although low in power with this dataset, successfully identified several GO terms and processes which were over‐represented by genes near mapped loci that were determined to be significantly affected by developmental stage or seawater *p*CO_2_ treatment (Table 2). In MBP larvae, significant effects of larval development were associated with GO terms for protein modification (GO:0036211), helicase activity (GO:0004386), anion binding (GO:0043618), lipid binding (GO:0008289), and organonitrogen compound metabolic processes (GO: 0016817). In wild groups, larval development was over‐represented by only two discrete functions: cell adhesion (GO:0016021) and “integral component of membrane” (GO:0016021). In all of these cases, low gene membership (*n* = 2–11 for MBP) and marginally significant *p*‐values (*p* > 0.05 in wild) and high multi‐functionality (MF) scores (0.217–0.901) warrant caution in interpreting GO categories.

**TABLE 2:** Functional enrichment: over‐represented functional groups for developmental stage (Stage) and seawater *p*CO_2_ treatment (Treatment) identified by Gene Score Resampling (GSR) in ErmineJ (Gillis et al., 2010) with a *p*‐value cutoff of <0.05

Type	Group	Name	Gene Ontology ID	# Genes	*p*‐value	Multi‐functionality (MF)	MF *p*‐value
Stage	MBP	Protein modification process	GO:0036211	2	0.031	0.434	0.022
		Helicase activity	GO:0004386	2	0.032	0.217	0.064
		Anion binding	GO:0043168	11	0.039	0.849	0.083
		Lipid binding	GO:0008289	2	0.044	0.243	0.034
		Organonitrogen compound metabolic process	GO:0016817	7	0.049	0.901	0.055
	Wild	Cell adhesion	GO:0007155	2	0.051*	0.143	0.048
		Integral component of membrane	GO:0016021	43	0.087*	0.016	0.287
Treatment	MBP	Phospholipid binding	GO:0005543	2	0.042	0.209	0.040
		Integral component of membrane	GO:0016021	52	0.049	0.011	0.313
	Wild	Membrane part	GO:0044425	45	4.10E−03	0.027	0.294
		Integral component of membrane	GO:0016021	44	6.00E−03	0.014	0.284
		Membrane	GO:0016020	48	9.50E−03	0.007	0.343

Note

Number (#) of genes indicates the total number of genes which were assigned to each group. Multi‐functionality (MF) is a measure, from 0–1, of estimated functional specificity, with higher values being more broad and smaller values more specific. “MF *p*‐value” is a scaling metric taking into account the adjusted *p*‐value in conjunction with multi‐functionality. Gene ontology IDs reported are the primary, but not exclusive, ID associated with this function. A comprehensive list of GO ID terms and genes assigned to each is found in Tables S4–S10. *These *p*‐values are only marginally significant (*p* > 0.05).

In contrast with larval development, seawater treatment effects seemed to have more consistent functional effects in MBP and wild larvae. In both groups, the over‐represented functions were associated with membranes: phospholipid binding (GO:0005543) in MBP larvae, two different types of membrane components in wild larvae (GO:0044425 and GO:0016020), and the GO term “integral component of membranes” (GO:0016021) was shared by both MBP and wild groups as a significant function. In contrast to the broad categorization of functions associated with larval development in ambient conditions (Stage), results for high *p*CO_2_ culture (Trt) were much more specific (lower MF scores; 0.007–0.209) and populated by more gene assignments (*n* = 2–52; Table 2). A full list of genes, associated GO terms, and the enrichment *p*‐values for these categories are found in the Supplemental Materials (File [Link], [Link]; Tables S4–S10).

## 4 DISCUSSION

These results contribute to a growing body of literature examining the genetic changes that oysters and other marine invertebrate species undergo during larval development as well as the potential genetic impacts of ocean acidification. Our findings highlight the complexity of the interaction between genetics, environmental stress, and larval development for these taxa. The disparate patterns of genetic change in domesticated and wild oyster stocks also provide an interesting insight into the potentially broad network of physiological functions that are crucial to successful larval development as well as the more specific set of functions that are affected by high *p*CO_2_/low Ω_arag_ environments.

### 4.1 Genetic changes intrinsic to larval development

Larval development was the single most impactful driver of genetic change in both MBP and wild larvae (“Stage” effects in Table 1). As postulated in our first hypothesis, improved recruitment success observed in MBP larvae, also corresponded with reduced genetic changes (~26% fewer SNPs with altered MAFs) compared with wild larvae. Larval survival is a significantly heritable trait in Pacific oysters (Ernande et al., 2003), and it is possible that this reduced genetic change reflects a domestication effect after five generations of rearing in hatchery conditions (Taris et al., 2006, 2007). It is also possible that the greater genetic diversity in wild oyster populations (Camara, 2011; Sutherland et al., 2020; Figure S3) may have resulted in a greater degree of genetic change, although we find little evidence to support this hypothesis across the scope of genetic changes in these two populations (discussed below).

Evidence of substantial genetic changes during larval development in oysters is not lacking. Previous studies have demonstrated that high numbers of negative alleles, or genetic load, in Pacific oysters from both naturally reproducing and selected stocks result in high larval mortality (~95%) and genotypic distortions in spat, relative to parental genotypes (Plough, 2012, 2018; Plough et al., 2016). It is difficult to directly compare the results from this study to previous estimates of genetic load in this species due to differences in marker type, cross–design, and genotyping approach. We expect that similar processes are taking place in this study but when using large composite populations and pooled DNA sequencing it is difficult to distinguish between signatures of genetic load and more general selection pressures for alleles generating intermediate fitness outcomes. Despite these limitations, our finding that ~34% of all markers analyzed here exhibited changes in MAF in at least one of the larval groups, and the majority did so in the absence of any apparent environmental stress, is compelling evidence to support the hypothesis that genetic load is a primary driver of the observed genetic changes in both larval groups. In this context, the reduced genetic changes in MBP larvae would suggest that selective breeding has successfully, but unintentionally, reduced the genetic load in this domesticated oyster stock, at least in the context of hatchery environments.

Traditional theories predict that purging of genetic load is favored by periods of inbreeding (Agrawal & Whitlock, 2012; Crnokrak & Barrett, 2002) but these models are largely predicated on the assumption that mutations are rare and recessive, and that breeding is random within the population. For Pacific oysters, by contrast, mutations are frequent (Penaloza et al., 2021; Zhang et al., 2012), many deleterious alleles may be additive or dominant in nature (Plough et al., 2016), and wild populations have been characterized as having chronically reduced effective population sizes due to “sweepstakes” reproductive success (Hedgecock & Pudovkin, 2011). These genetic factors and population dynamics have been proposed as the primary mechanisms by which high genetic load is maintained in this species (Plough, 2016). While overall rates of mutation in oysters are not likely to have been affected by selective breeding, the reduction of inbreeding in MBP stocks may have allowed for a more uniform distribution of negative alleles among breeding families, thereby reducing the compounding effect of multiple loci with negative alleles within individual parents. Alternatively, dispersed load may also allow for some degree of purifying selection in MBP stocks by reducing the effects of genetic drift. These two hypotheses—domestication to hatchery conditions and reduced effective genetic load—are not mutually exclusive and both are plausible ways to explain the findings in this study.

The extent to which the oysters sampled from the naturally recruited population in Willapa Bay, WA for this study represent the broader genetic diversity of Pacific oysters in the region is not known. Previous work has suggested that temporal changes in genetic composition are substantially greater than regional heterogeneity for these stocks (Sun & Hedgecock, 2017). It is possible that for naturally recruited oysters on the West Coast of North America, genetic changes during larval development may vary significantly between cohorts spawned from parents collected from different regions or generations. Further research investigating the genetic load of oysters in wild populations and structured breeding programs would provide useful insights to clarify the relative influence of mutation rate and population dynamics in the maintenance of negative mutations in these and other species that share similar early life‐history characteristics.

### 4.2 Genetic changes in oyster larvae in response to acidified seawater

Similar to the genetic effects of larval development, we found that high *p*CO_2_ seawater also had a markedly greater impact (>2× more SNPs with changed MAFs) on larval pools from wild than MBP broodstock, supporting our second hypothesis. The genetic effects of seawater treatment on larval survival also interacted extensively with larval developmental processes, in both compounding and contrasting ways. In wild larvae, ~1/3 of all SNPs with MAFs affected by high *p*CO_2_ seawater were also additively impacted by developmental stage (Stage + Trt; Table 1, Figure 2). This type of interaction is consistent with the findings of Plough (2012), who reported that, during larval oyster development, environmental stress (diet quality in that study) doubled the number of detected loci with negative alleles and increased both strength of selection and dominance of these variants. In MBP groups, a very small number of SNPs had MAFs affected by these processes in tandem (*n* = 7), which also points to the possibility of adaptation to hatchery conditions or reduced genetic load in these selected lines.

In addition to the additive effects of *p*CO_2_ stress and larval development, there was also an abundance of loci with alleles which exhibited interactive effects between these two processes (Stage * Trt; Table 1, Figure 2). Positive interactions indicate a consistent and magnified effect of *p*CO_2_ stress on allele frequency changes across larval development and negative interactions point to antagonistic effects. Among the significant interactions, ~89% and ~75% in MBP and wild groups, respectively, were negative interactions (Figure S5). For these loci, the differences in mean allele frequency between ambient and high *p*CO_2_ environments at day two were reversed by day 22. This indicates that there is likely a disconnect between some of the genetic effects on larval survival early in development and those contributing to later larval fitness under acidified conditions. This is evidence to suggest that genes underpinning larval survival in high *p*CO_2_ seawater may be antagonistically pleiotropic and incur life‐history trade‐offs over the entire developmental period (i.e., Sgro & Hoffmann, 2004). Just as larval oysters display phenotypic impacts to acidified seawater that are stage‐specific (Durland et al., 2019; Waldbusser et al., 2015b), it also appears that the genetic components of larval fitness also vary considerably across developmental stages and be subject to balancing selection (Durland et al., 2021). More generally, these results suggest caution is warranted when evaluating the phenotypic or genetic impacts of environmental stressors (such as OA) on larvae at a discrete time point or developmental stage and then extending the scope of inference to the overall performance of the species or population (e.g., Wittmann & Pörtner, 2013).

In this study, high *p*CO_2_ conditions did not significantly reduce the survival or development of larvae in either MBP or wild oyster groups (Figure S4). These findings are somewhat contradictory to the widely reported negative effects of high *p*CO_2_/low Ω_arag_ conditions on larval growth and survival during early stages (e.g., Kurihara et al., 2007; Waldbusser et al., 2015a). Larvae reared in high *p*CO_2_ conditions in this experiment had adverse OA effects on larval development at day 2, similar to those reported in previous studies (Figure S4), but these effects diminished during later veliger stages and did not apparently reduce overall survival or recruitment success in either group (see Durland et al., 2019 for more details).

It is interesting to note that we detected genetic changes in high *p*CO_2_ cultures despite a lack of significant impact on overall larval mortality. If the observed genetic changes in response to OA conditions were accompanied by increased mortality, one could infer that these changes were the signatures of selection in this stressful environment. When genetic differences arise in the absence of additional mortality, however, it implies that altered allele frequencies for these SNPs are attributable to changes both in the ambient and high *p*CO_2_ larval groups equally. This indicates that many of the genetic changes that are attributable to OA effects in this study represent fitness trade‐offs for larvae: providing improved survival in one environment and reduced performance in the other. This finding is similar to those of Bitter et al. (2019), who observed a “reshuffling” of standing genetic diversity in larvae of the Mediterranean mussel (*Mytilus galloprovincialis*) reared in low and high *p*CO_2_ seawater with no significant difference in survival. In the current study, wild larvae had ~230% more SNPs with altered allele frequencies in response to seawater treatment, suggesting genetic trade‐offs were markedly greater in this population than in larvae from domesticated oyster stocks.

The extent to which selection pressures and physiological trade‐offs assist or constrain the long‐term adaptive capacity of marine organisms that inhabit variable environments, such as oysters, remains one of the pressing questions for conservation biology in the era of climate change (Kelly & Hofmann, 2013). These findings support previous studies that have highlighted the potential of marine invertebrates to adapt to climate change stressors like OA (e.g., Bitter et al., 2019; Brennan et al., 2019; Pespeni et al., 2013). Our results also suggest, however, that the inherent aspects of larval development such as genotype‐dependent mortality and genetic load may complicate the adaptive response of these species in ways that vary between even closely related populations. The extent to which background genetic mechanisms dictate the variance in resilience or adaptive potential of vulnerable species, such as oysters (e.g., Parker et al., 2010), deserves attention in order to improve our understanding of how climate change affects populations of marine organisms.

### 4.3 Disparate loci under selection in oyster populations

The most unexpected finding from this study was not that there was a quantitative difference in genetic changes between MBP and wild larvae but that these changes were largely unique to each group and category of effect (Table 1). This result seems biologically counter‐intuitive and would be most plausibly attributed to differences in background genetic diversity between the groups. If there was a strong disparity in starting allele frequencies between MBP and wild larval populations, for example, an excess of rare or private alleles in one population, it would be unsurprising that different SNPs exhibited changes over development. While wild larvae may have been slightly more genetically diverse (Figure S3), direct comparisons of beginning allele frequencies (day 2) between the groups (Figure S6) demonstrate that only ~10% of all the SNPs with MAFs affected by “Stage” in one of the groups had little or no variance in the other. Additionally, the few shared “Stage” SNPs (*n* = 4) started with MAF values of <5% in both groups, indicating that small changes in low variance SNPs were not overtly obscured by our analytical methods. Overall, we find insufficient evidence to conclude that differences in genetic diversity adequately explain the disparate genetic changes associated with larval development in MBP and wild populations.

Another possible way to explain the disparate genetic changes that we observe is that pooled DNA samples inaccurately represent the allele frequency of a population when individuals within the sampled pool are unequal in size (i.e., differential contribution of DNA). This technical artifact may have arisen in day 22 samples where spat were significantly larger than pediveliger larvae. MBP groups had more and larger spat than wild larval populations (Figure S4) and, thus, would potentially be more susceptible to this type of error. The significantly greater amount of genetic change in wild groups, however, suggests that any “skew” effects of this nature had less overall impact on the major findings than the differences between the oyster stocks themselves. More generally, the accuracy of poolseq is dependent upon numerous factors including pool size, library preparation, and sequencing depth. In this study, we followed “best practices” for data curation (e.g., pool size >40, >50× coverage, reads with single mapping; Schlötterer et al., 2014) and leveraged a statistical analysis on five biological replicates in this experiment to account for remaining technical variation. This conservative approach sacrificed some power of the dataset (number of SNPs) in favor of greater reliability. Given the significance of the results we observed, however, it seems unlikely that a “larger but weaker” dataset would lead to significantly different findings from those presented here.

In addition to differences in genetic diversity between the populations or variance arising from a poolseq approach, it could also be proposed that linkage disequilibrium decay may account for the stark contrasts in the observed genetic changes. With a short‐read RADseq approach, one of the analytical assumptions is that polymorphisms (SNPs) are associated with, or “linked” to, causal genetic variants uniformly across populations. In two distantly related populations, the stochastic processes of meiotic recombination and drift may reduce the frequency of this association, known as linkage disequilibrium decay (Baird, 2015). For the MBP and wild broodstocks, which are separated by ~5 generations, we can expect that some SNP‐gene associations are no longer consistent between the populations. Even so, gene variants that are responsible for strong selective sweeps, such as those predicted to occur with deleterious alleles and genetic load, should share a selection signature at concordant genomic regions (e.g., Plough et al., 2016). When we compare Manhattan plots for “Stage” or “Trt” effects across the 10 linkage groups of the genome in MBP and wild larval groups (Figure 3, Files S3 and S4), we find abundant outlier regions, but no obvious patterns or clusters in common. This implies that either concordant peaks are absent and that genetic effects are truly different for each group or that concordant peaks are so abundant that they are not easily identified. The latter explanation is perhaps more likely, but given that the actual SNPs with changing allele frequencies are largely unique to each population, this rationale would require extensive genetic differentiation between these groups and substantial linkage disequilibrium decay to account for this disparity. Mapped markers only account for ~25% of all SNPs analyzed with the GLM, and the coarse resolution on the genomic scale limits our ability to distinguish between truly unique genetic effects, misinterpretations due to linkage decay, or other structural variation that is undetected. In any scenario, these findings are surprising and point to a level of genetic complexity for these traits that have not previously been described, to our knowledge, in any other species. In an applied sense, these results suggest that genetic markers for larval fitness, such as employed in QTL analyses (e.g., Wang et al., 2016), may vary substantially and have low predictive power when a single marker set is used across different breeding stocks or populations.

With the inability to directly tie observations of disparate genetic changes to a single causal mechanism, we must allow for the possibility that several of these factors contributed independently to these findings. Even so, we are unable to validate the first part of our third hypothesis—that patterns of genetic change in each group share a similar identity. Determining the biological cause of these dissimilar genetic identities would be valuable in understanding adaptive responses as well as the challenges in using molecular genetic tools to identify consensus effects in populations of marine invertebrates.

### 4.4 Functional analyses of loci under selection

Although the genetic changes we found were largely specific to each group, one remaining explanation to account for this disparity is that different genetic vulnerabilities were detected which shared a similar functional role. We found that for larval development under ambient conditions, no specific gene ontology categories were uniquely over‐represented with compelling statistical strength (Table 2). Given the notable quantity of SNPs with significantly changed allele frequencies throughout development in both groups, a lack of functional consensus likely reflects the breadth of physiological functions that are critical to survival during larval development in marine invertebrates (De Wit et al., 2018; Huan et al., 2012; O’Donnell et al., 2010). This finding is similar to those of Pespeni et al. (2013) and Bitter et al. (2019) who reported similar substantial genetic changes in larval invertebrates reared in ambient seawater without attribution to discrete functional categories.

Genes associated with changes in allele frequencies due to high *p*CO_2_ culture conditions appear to be significantly over‐represented by GO categories concerning aspects of membrane function and structure in both larval groups (Table 2). In fact, all of the genes which were attributed to GO categories over‐represented in MBP larvae were also found in the corresponding list for wild larvae. This is compelling evidence to support the second half of hypothesis #3: that the genes under selection in both groups share a common function. In both groups, genes corresponding to amiloride‐sensitive cation channels (CGI_10027240), XK‐related proteins (CGI_10026940), and Aquaporin‐4 (CGI_10022547) were significantly over‐represented. These functions all play important roles in controlling the transport of cellular materials such as peptides, amino acids, and ions across membranes and in maintenance of osmotic gradients. A complete list of genes, their GO categories, and *p*‐values which contributed to the functional enrichment analyses can be found in the Supplemental Material (Tables S9–S10). The more extensive functional overlap between the two larval groups, despite having very few SNPs changing in common, may be a product of a more generous statistical approach in these analyses (see Methods) compared to the GLM and FDR thresholds. Alternatively, it may also suggest the presence of gene duplicates for these functions, which are expected to be abundant for stress‐related traits in this species (Penaloza et al., 2021; Zhang et al., 2012).

Cellular membranes are predicted to play an especially important role for bivalve larvae reared in acidified environments by maintaining ion homeostasis within cells (Frieder et al., 2018; Pan et al., 2015) as well as modifying Ω_arag_ in the extracellular space at the site of calcification (De Wit et al., 2018; Ramesh et al., 2017). Numerous other studies have evaluated the effect of simulated OA conditions on the proteome and transcriptome of larval and adult bivalves (e.g., Dineshram et al., 2012; Hüning et al., 2013; Liu et al., 2020; Wei et al., 2015). These previous analyses have demonstrated that the functional scope of genes affected by simulated OA conditions is broad but ion regulation, homeostasis, and maintenance of cellular membranes are recurrently overabundant. Our results extend this inference and suggest that genetic variation for these aspects of physiology in larval oysters is targets of selection in high *p*CO_2_ environments. These findings are in agreement with similar studies with urchins and mussels (Brennan et al., 2019; Pespeni et al., 2013) which reported changes in genetic variation for genes related to ion regulation and membrane structure in urchin larvae when reared in high *p*CO_2_ conditions.

## 5 CONCLUSIONS

We have found that genetic changes in larval oyster populations across developmental transitions are profound, abundant, genome‐wide, and likely associated with numerous aspects of larval physiology. This result is broadly consistent with the hypothesis that genetic load is a primary driver of larval mortality in oysters as proposed by Plough et al. (2016). In this work, however, we additionally demonstrate that larvae from selectively bred stocks (MBP) showed significantly less genetic change than their wild counterparts. This is evidence to suggest that MBP lines have been domesticated to hatchery conditions or that these stocks have a reduced load of negative alleles in the breeding population, although these explanations are not mutually exclusive. Additionally, the changes in allele frequencies taking place in each larval group were largely dissimilar, potentially indicating either substantial differentiation between these recently related populations or significantly different targets of selection pressures during larval development.

Despite a lack of a significant impact of high *p*CO_2_ seawater on overall larval survival or metamorphosis success in this study, culture in acidified seawater did, nevertheless, have significant impacts on the genetic makeup of larval groups by the time of metamorphosis. Overall, acidified seawater resulted in changes of allele frequencies at >2× more SNPs in wild than MBP larvae, indicating that trade‐offs for survival in acidified sweater are significantly reduced for these selected lines, at least in hatchery settings. The predicted genes in association with markers affected by high *p*CO_2_ seawater were disproportionately attributable to functional categories concerning membrane structure and function in both groups. This finding reinforces the hypothesis that cellular membrane activity and the regulation of ionic gradients are critical physiological functions necessary for the survival of larval oysters in acidified seawater.

We found significant interactions between genetic determinants of larval survival and those specific to acidification stress, which complicate predictions of the adaptive capacity of oysters in natural settings. With population‐specific responses and strong temporal (developmental) effects on allele frequencies at numerous loci, genetic marker‐based approaches for predicting breeding values for larval traits, such as fitness in high *p*CO_2_ seawater, may be difficult to employ and have limited applicability among different populations. The genetic changes in larval oysters during development appear to be complex and dynamic, and further work is necessary to resolve how these changes are affected by the myriad abiotic stressors found in natural environments.

## CONFLICT OF INTEREST

None declared.

## Supporting information

Fig S1‐S6

Table S1‐S10

Supplementary Material

Supplementary Material

## Data Availability

Bioinformatics quality statistics: File S1; Table S3. Full output of functional enrichment analyses: File [Link], [Link]; Tables S4–S8. Gene annotations for membrane functions: Tables S7–S8. FASTQs for all samples and tabular data for all 1288 SNPs used in these analyses are uploaded to Dryad at: https://doi.org/10.5061/dryad.mgqnk98vw.
