# Supplementary material for: Larval development in the Pacific oyster and the impacts of ocean acidification: Differential genetic effects in wild and domesticated stocks
Source: Evol Appl. 2021 Aug 26;14(9):2258–72. doi: 10.1111/eva.13289 (PMC8477599; doi:10.1111/eva.13289)
Supplement: Supplementary file 1 — Fig S1‐S6 [file EVA-14-2258-s003.docx]

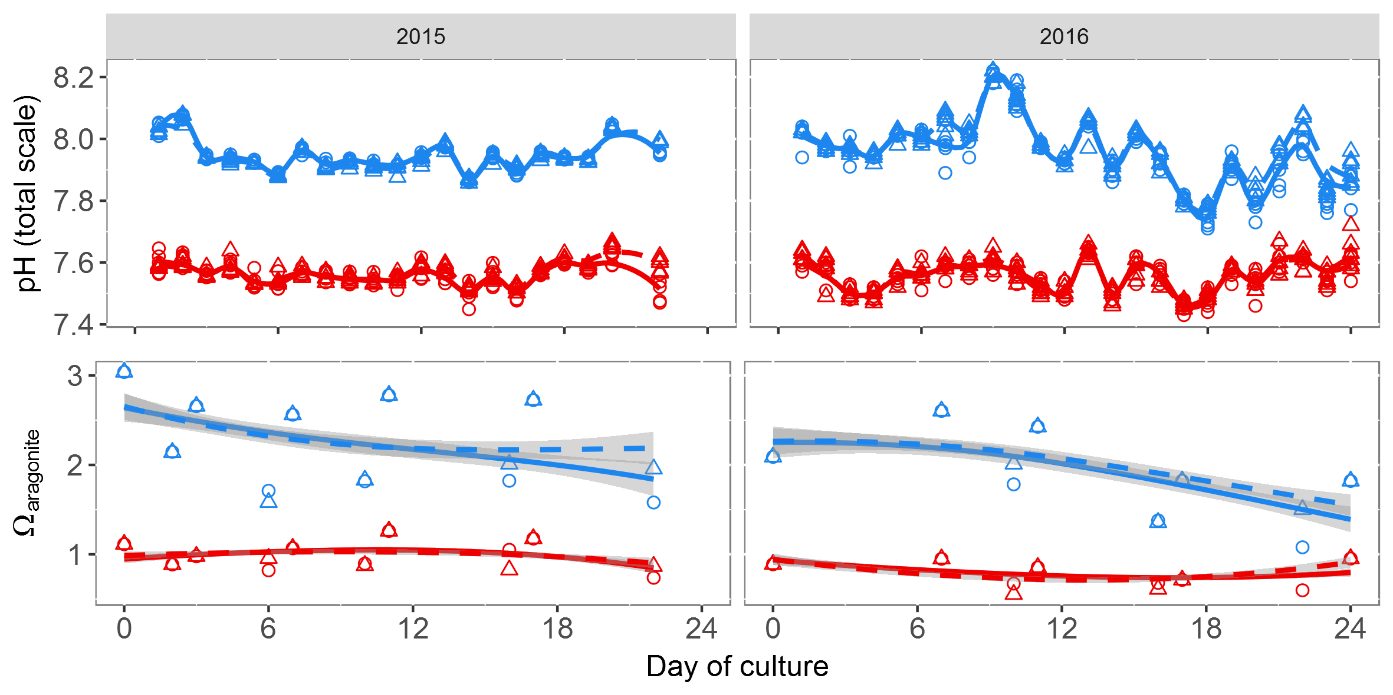

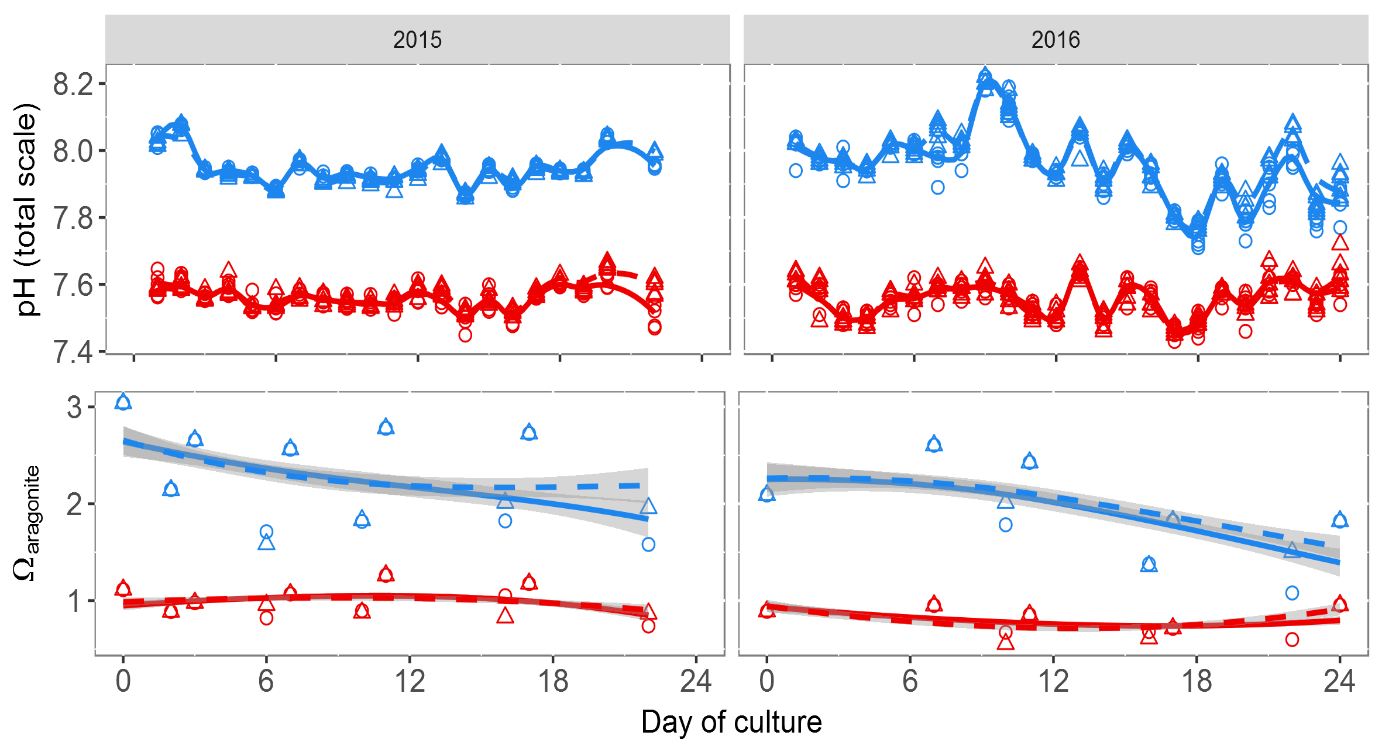

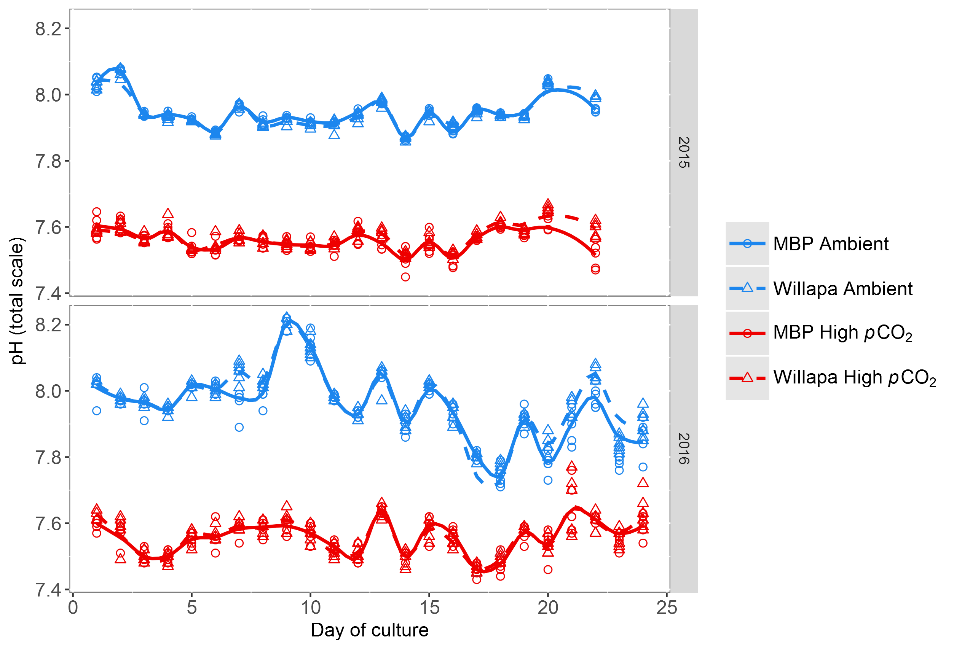

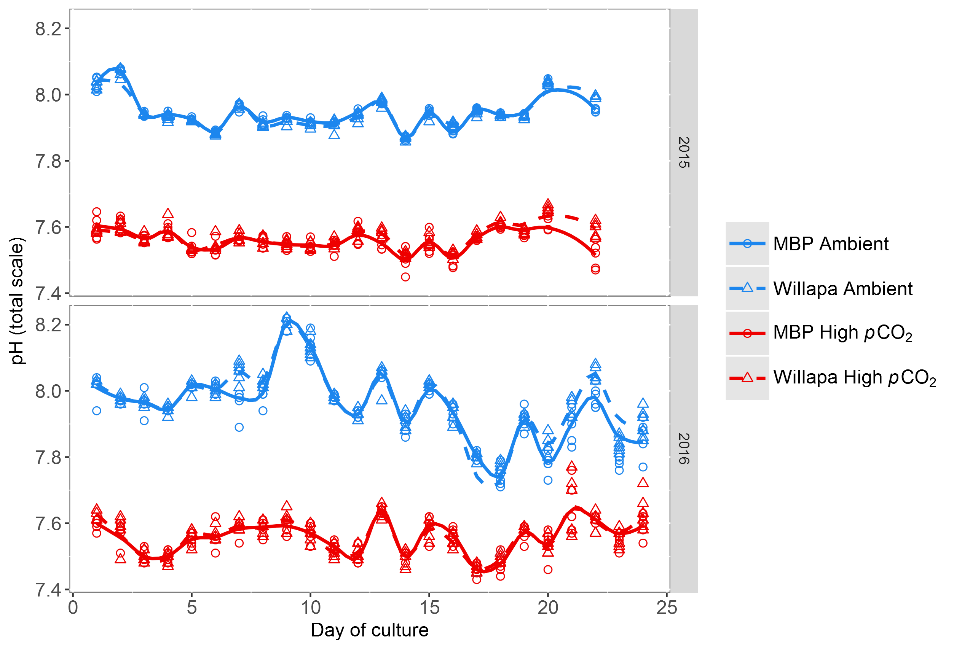


**A**

**BA**

MBP ambient

Wild ambient

MBP high *p*CO_2_

Wild high *p*CO_2_

Day of culture

**Figure S1**: Summary of pH (A) and saturation state of aragonite (Ω_aragonite_; B) across the 22 day experimental period for MBP (solid lines) and Wild (dashed lines) larvae reared at ambient (blue) and high (red) *p*CO_2_ levels

**
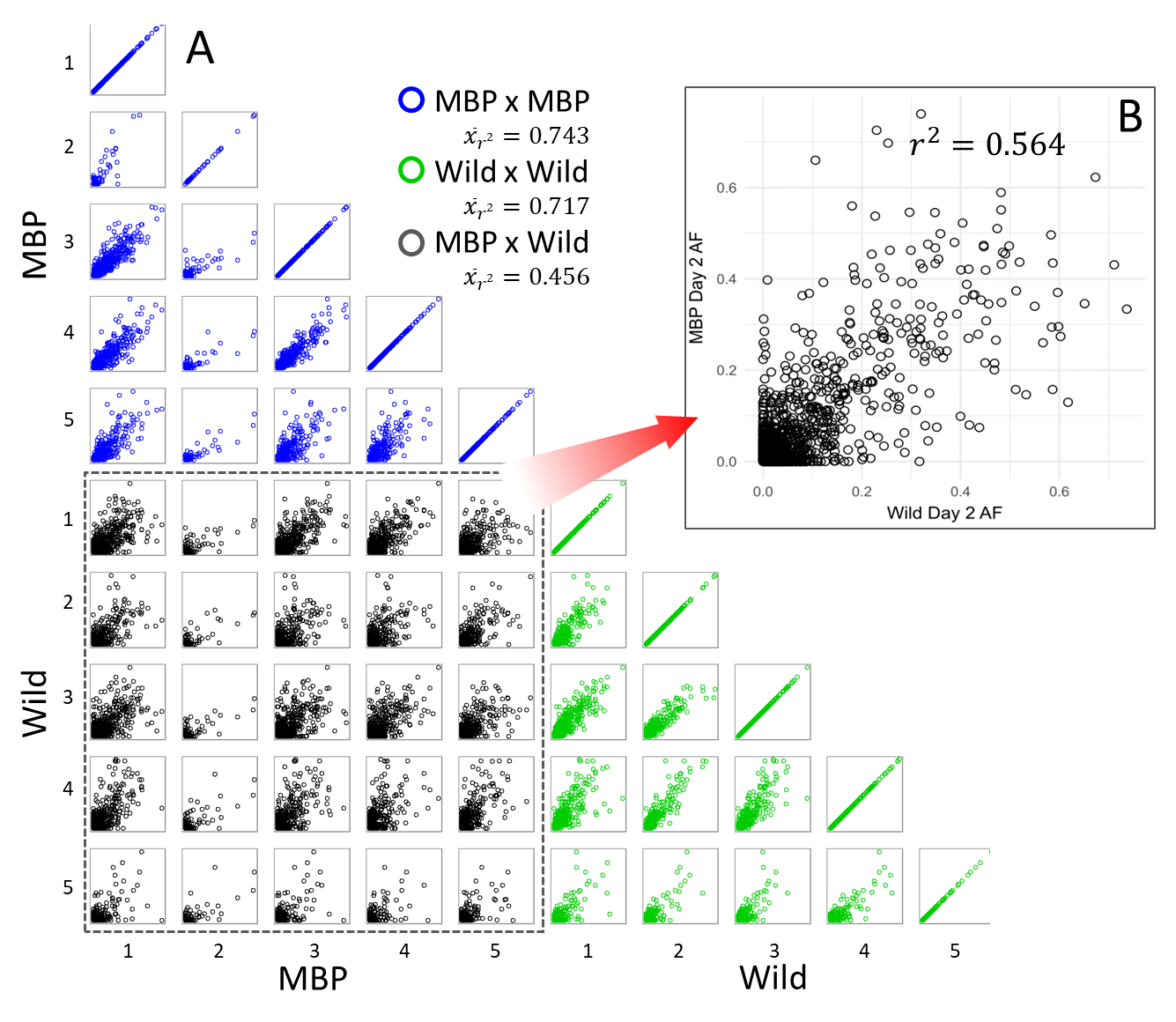
Figure S2**: Comparing pooled allele frequencies between replicates and groups. A) Day 2 MAF for MBP (blue) and wild (green) for each replicate are compared to the MAF of each of the nine other replicates (both within the group and outside it). B) mean MAF from each group (MBP and wild) compared to each other.


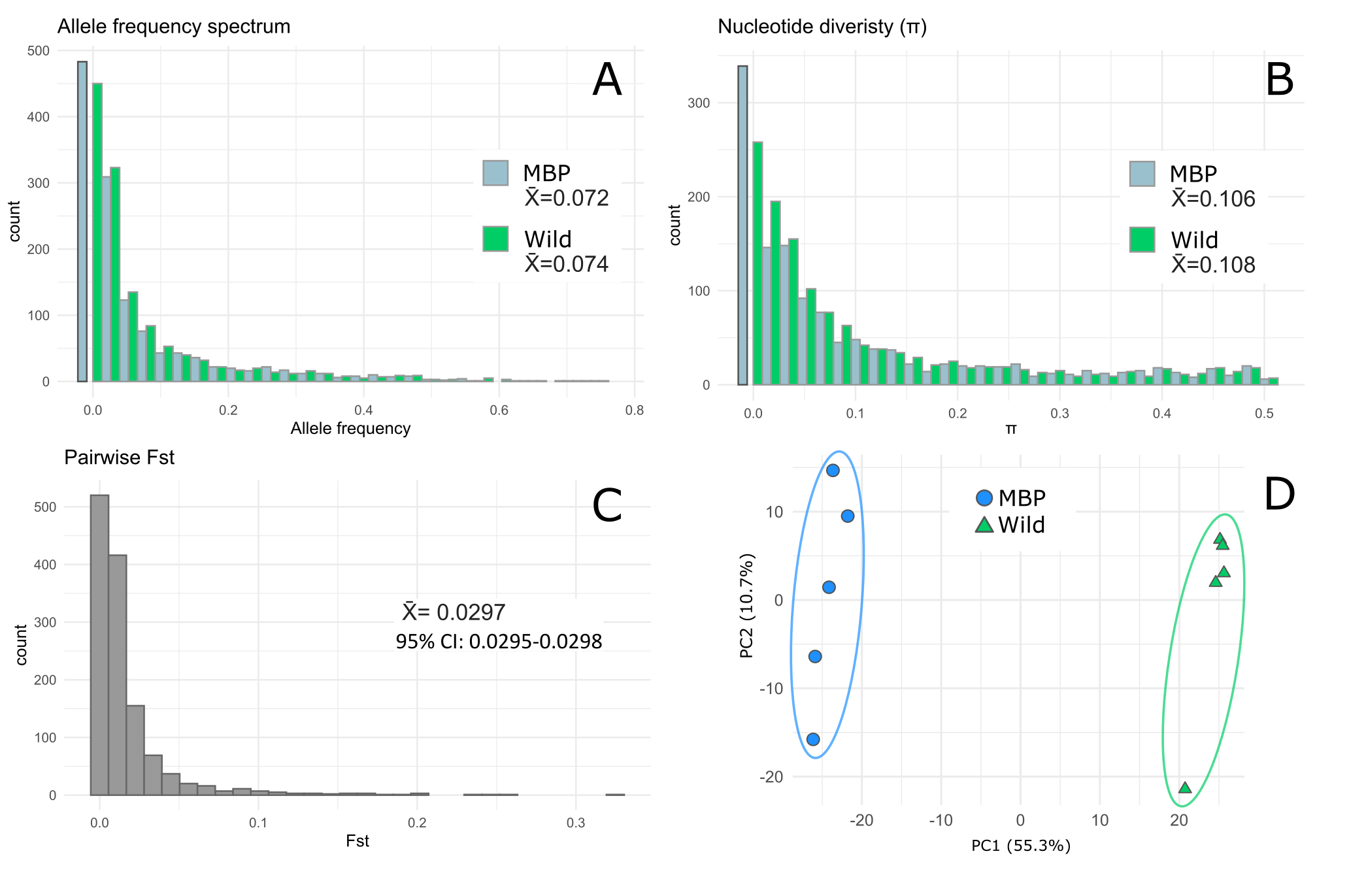


**Figure S3**: Genetic diversity comparisons between MBP and wild larval oyster pools. A) Allele frequency spectrum of all SNPs; B) Nucleotide diversity for all SNPs; C) Pairwise Fst; and D) PCA plot. The separated blue bar on the left of the column charts (in panels A and B) highlights SNPs with no minor alleles detected in MBP larval pools. Nucleotide diversity was calculated based on methods from Begun et al. (2007). All analyses used mean allele frequencies of SNPs from Day 2 larval pools, without discarding rare variants (e.g. < 1% MAF).


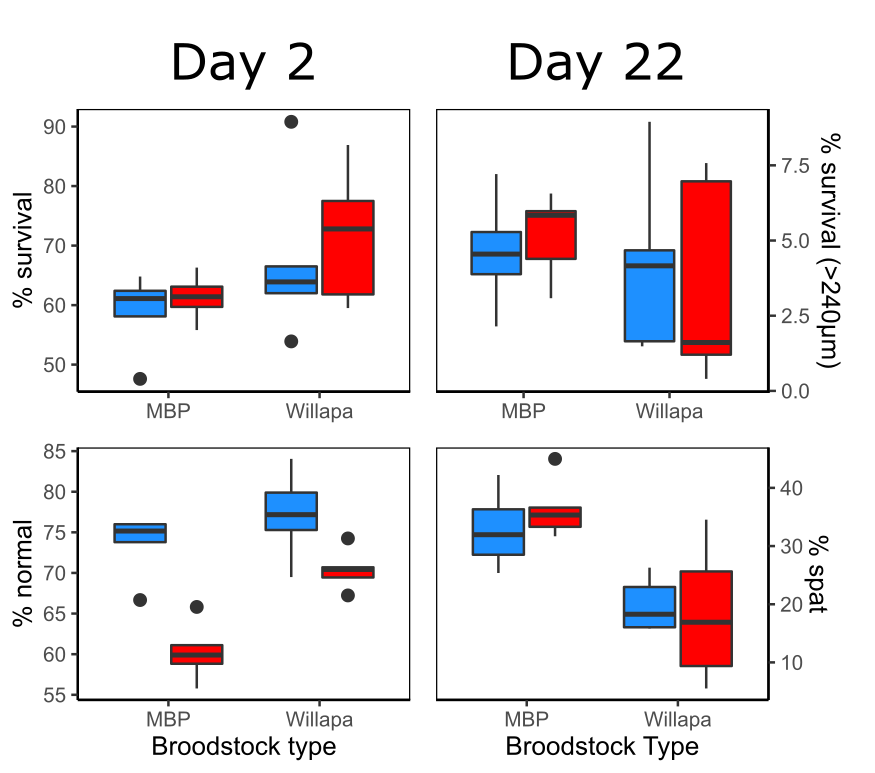


D

C

A

B

**Figure S4**: Survival of all larvae at day 2 (A) and 22 (B) post fertilization and percent ‘normal’ D-hinge larvae at day 2 (C) and percent spat at day 22 (D) for MBP and wild groups reared in ambient and high *p*CO_2_ conditions. Mean survival and size of each group and treatment, along with statistical analyses can be found in Durland et. al.,(2019); Table S2 (means), S3 (Cumulative survival), S5 (Day 2 survival), S6 (Day 2 % normal), and S15 (% spat).


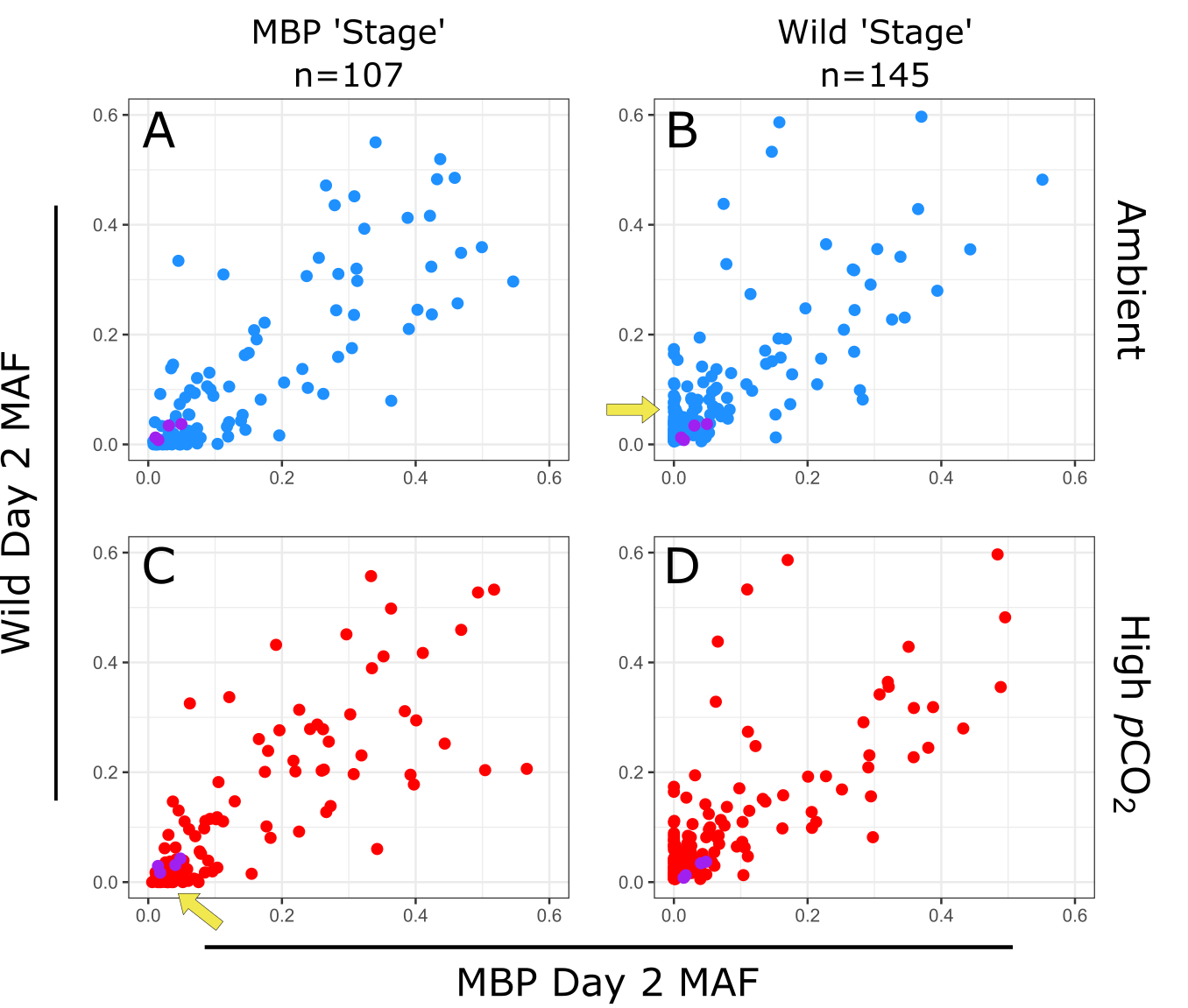


**Figure S5**: ‘Starting’ minor allele frequency comparisons at Day 2 for MBP and Wild larvae in Ambient (blue; A&B) and High *p*CO_2_ (red; C&D) conditions. Figures (A) and (C) represent n=107 SNPs which were found significantly changed by larval development (‘Stage’) in MBP groups. Figures (B) and (D) represent n=145 ‘Stage’ SNPs for Wild larval groups. There are only n=4 SNPs significantly changed by ‘Stage’ in both groups (purple). The yellow arrows indicate SNPs for which a low minor allele frequency in one group may obscure the detectable change in frequency in the other with comparably higher starting MAF.


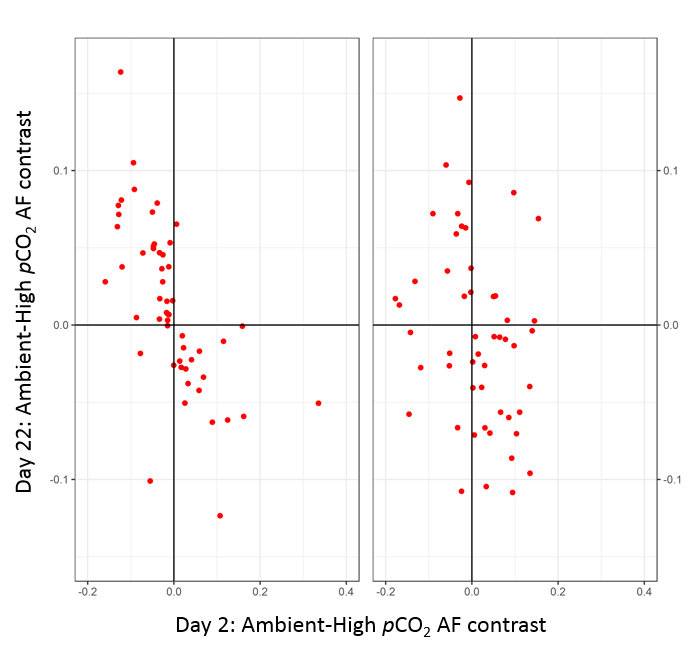


Wild

MBP

**Figure S6**: Contrasts in mean allele frequency (AF) for significant ‘Stage * Trt’ loci between ambient and high *p*CO_2_ seawater at day 2 (x-axis) and day 22 (y-axis) post fertilization. Per-locus contrasts in AF are calculated as the mean allele frequency of acidified cultures minus that of ambient replicates. Approximately 89% of MBP and 75% of wild loci (points) have negative interactive effects, represented by falling in the upper left and lower right quadrants of the plots.

Begun, D. J., Holloway, A. K., Stevens, K., Hillier, L. W., Poh, Y. P., Hahn, M. W., . . . Langley, C. H. (2007). Population genomics: whole-genome analysis of polymorphism and divergence in Drosophila simulans. *PLoS Biol, 5*(11), e310. doi:10.1371/journal.pbio.0050310
