# Supplementary figures and images for: Larval development in the Pacific oyster and the impacts of ocean acidification: Differential genetic effects in wild and domesticated stocks
Source: Evol Appl. 2021 Aug 26;14(9):2258–72. doi: 10.1111/eva.13289 (PMC8477599; doi:10.1111/eva.13289)

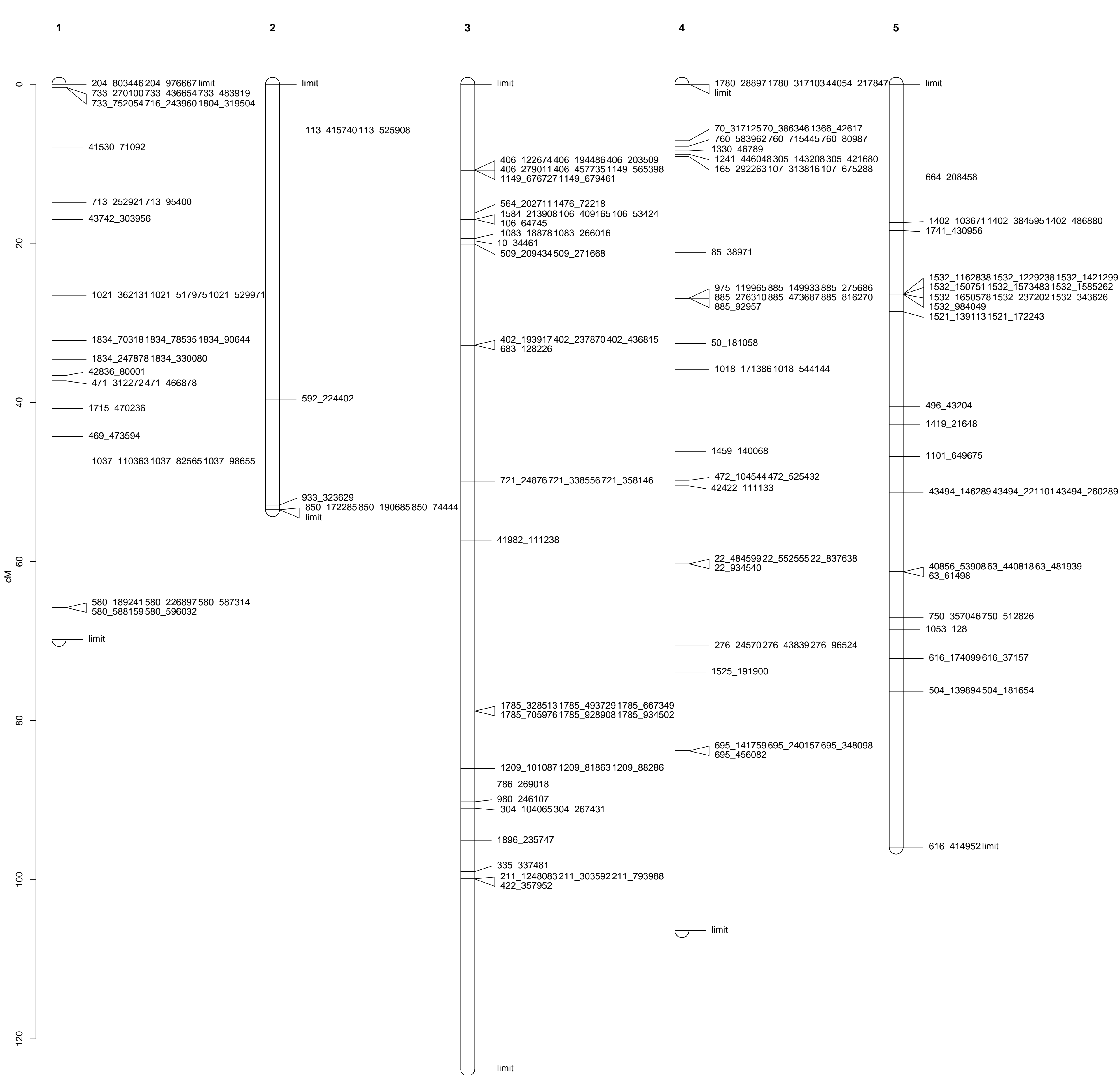

Supplement: Supplementary file 3 — Supplementary Material [file EVA-14-2258-s001.pdf]

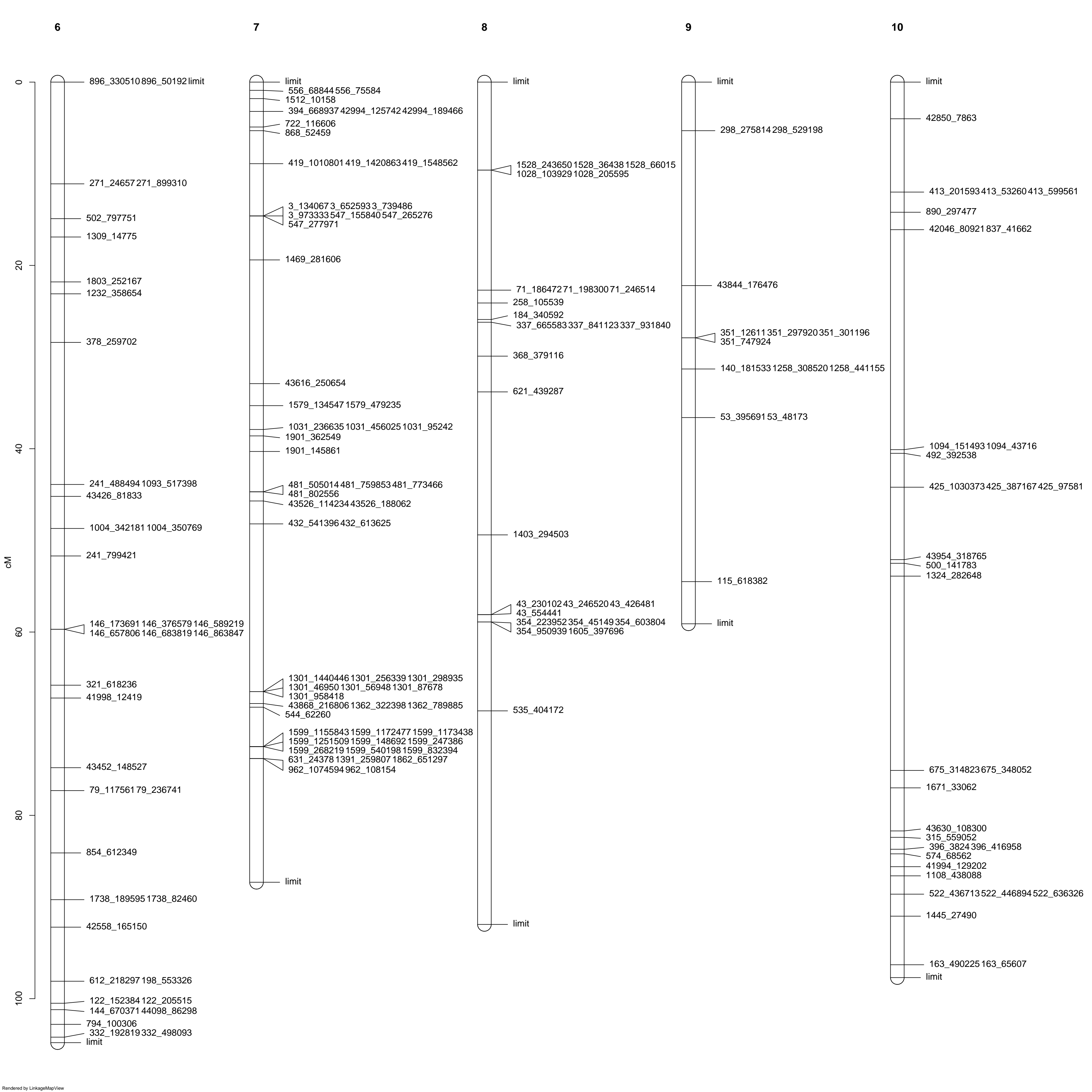

Supplement: Supplementary file 4 — Supplementary Material [file EVA-14-2258-s002.pdf]
